# Supplementary material for: Predicting human body composition using a modified adaptive genetic algorithm with a novel selection operator
Source: PLoS One. 2020 Jul 16;15(7):e0235735. doi: 10.1371/journal.pone.0235735 (PMC7365447; doi:10.1371/journal.pone.0235735)
Supplement: Attachment — Submitted filename: Response to Reviewers.docx [file pone.0235735.s002.docx]

**Original Manuscript ID:** PONE-D-19-20446

**Original Article Title: “Predicting human body composition using a modified adaptive genetic algorithm** ”

**To:** PLOS ONE Editor

**Re:** Response to reviewers

Dear Editor,

Thank you for allowing a resubmission of our manuscript, with an opportunity to address the reviewers’ comments.

We are uploading (a) our point-by-point response to the comments (below) (response to reviewers), (b) an updated manuscript with yellow highlighting indicating changes, and (c) a clean updated manuscript without highlights. In addition, we proofread the previous simulation results and updated the data in Table 5 and the ethics statement was added to the Method section of the manuscript.

We tried our best to improve the manuscript and made some changes in the manuscript.  These changes will not influence the content and framework of the paper. We appreciate for Editors/Reviewers’ warm work earnestly, and hope that the correction will meet with approval.

Best regards,

<Bo Chen> et al.

**Reviewer#1, Concern # 1:** Line 287 the authors mentioned w1-w11 but the formula only show up to w9, where are w10 and w11?

**Author response:** Thank you for pointing out these problems. This is a writing error by the author. In fact, there are only 9 regression coefficients in the fitted model. It has been modified.

**Author action:** We updated the manuscript by changing $\omega_{1}\text{\textasciitilde}\omega_{11}$ to $\omega_{1}\text{\textasciitilde}\omega_{9}$ and replacing $\omega=[\omega_{1},\omega_{2},\omega_{3},\omega_{4},\omega_{5},\omega_{6},\omega_{7},\omega_{8},\omega_{9},\omega_{10},\omega_{11},b]$ with $\omega=[\omega_{1},\omega_{2},\omega_{3},\omega_{4},\omega_{5},\omega_{6},\omega_{7},\omega_{8},\omega_{9},b]$.

**Reviewer#1, Concern # 2:** Please explain how alpha and beta values are determined.

**Author response:** Thank you for reviewing our article. The alpha and beta values are determined based on the results of the simulation experiment. It can be seen from the supplementary simulation figure that when the ratio of alpha to beta is 2: 1, the mean square error of the prediction model is the smallest. Therefore, the value of alpha is one third and the value of beta is two thirds.

**Author action:** We updated the manuscript by adding a simulation figure 8 as well as its discussion in the results and discussion section.

**Fig 8. Mean square error of different ratios of** $\alpha$ **and** $\beta$**.**

**Reviewer#1, Concern # 3:** The english need to be improved.

**Author response:** Thank you for pointing out these problems. We have tried our best to check and modify the errors in the manuscript, including misspellings of words, grammatical errors and inappropriate expressions and so on. There are many changes which have been highlighted in yellow in the manuscript.

**Author action:** We did our best to correct the errors in the article to improve the english.

**Reviewer#1, Concern # 4:** Include a statistical test to show significancy between the different algorithms that were compared.

**Author response:** Thank you for pointing out these problems. This paper has compared and analyzed the importance of different algorithms from the aspects of error, correlation coefficient and running time. In addition, for further analysis, we have added simulations comparing MAGA with support vector regression algorithm (SVR), artificial neural network algorithm (ANN) and traditional regression method (TRM) to show significancy. The significancy of the algorithms is analyzed as follows:

(a) As can be seen from Figure 10, when compared with GA, AGA and IAGA, the MAGA has a stronger optimization ability for finding the optimal weight set with highest fitness (0.9921) and avoiding premature convergence to the local optimum.

**Fig 10. Comparative analysis of the fitness of different models.**

(b) As can be seen from Figure 10, the MAGA model fitness has been increasing during population evolution, but the fitness of other algorithms fluctuates during the ascent. Therefore, the MAGA has better robustness during the ascent.
(c) As can be seen from Figure 12, Table 4 and Table 5, although TRM, GA and AGA models have low accuracy, thier training and running time is shorter. What is more, the results demonstrate that the relative error curve of the MAGA model has minimal fluctuations, the average relative error and the mean square error is the smallest and the correlation coefficient is the largest, meaning that the prediction model’s accuracy has been improved by MAGA. What’s more, the MAGA model with satisfactory training and running time has higher efficiency than IAGA, SVR and ANN models. In general, the significancy of the proposed algorithms can be shown from the simulations.

**Fig 12. Comparison of relative errors between actual values and predicted values obtained by different models.**

**Table 4. Comparison of the training time for different models.**

| Model | Maximum fitness degree | Training time(s) |
| --- | --- | --- |
| GA | 0.5642 | 26.82 |
| AGA | 0.8074 | 28.23 |
| LAGA | 0.9532 | 28.94 |
| MAGA | 0.9921 | 28.58 |
| SVR | - | 30.67 |
| ANN | - | 29.89 |
| TRM | - | 25.46 |

**Table 5. Comparison of the performance of different models.**

| Model | Average relative error (%) | Mean square error | Correlation coefficient | Running time(s) |
| --- | --- | --- | --- | --- |
| AGA | 0.2185 | 6.27 | 0.684 | 2.66 |
| LAGA | 0.1045 | 2.72 | 0.929 | 2.97 |
| MAGA | 0.0500 | 1.30 | 0.982 | 2.84 |
| SVR | 0.0873 | 2.56 | 0.927 | 3.02 |
| ANN | 0.1058 | 2.85 | 0.920 | 2.95 |
| TRM | 0.2348 | 6.73 | 0.596 | 2.49 |

**Author action:** We updated the manuscript by adding simulations and discussion to show the significancy of the algorithms.

**Reviewer#1, Concern # 5:** How were defined the number of samples selected as training and test sets? are these amounts enough? Why didn't the authors use cross validation? Where was the model implemented and tested (computer characteristics, software, etc.)?

**Author response:** Thank you for reviewing our article. The answers to your questions are as follows:

(a) The number of samples in the training set is generally at least greater than 50% of the total number of samples. To make the model better generalization ability, we selected 200 training samples and the remaining 20 samples are test samples.
(b) It can be known from the comparative simulation results that the proposed prediction model has more excellent performance than other models when we used this number of samples. Therefore, these amounts are enough.
(c) Due to the high computational cost of cross validation, we utilized random sampling method instead of cross validation, which can reduce the calculation time. In the future research, we will use cross validation to further analyze the model performance.
(d) The model was implemented and tested in Matlab R2016a and Python 3.5.4 software environment. The computer processor model used in this study is Intel (R) Core (TM) i7-6500U. What’s more, the computer's main frequency and RAM are 2.50GHz and 8G, respectively.

**Reviewer#2, Concern # 1:** Background missing. Clarity of presentation is needs to improved via clearly specifying objectives, focus and motivation of the proposed work.

**Author response:** Thank you for pointing out this problem. We added background information and clearly specified objectives, focus and motivation of the proposed work.

**Author action:** We updated the manuscript by adding background information and clearly specifing objectives, focus and motivation of the proposed work in the paragraphs 7, 8 and 9 of the introduction section. The added and modified content is as follows:

(a) More considerably, although surprisingly it has stronger optimization capability and high convergence efficiency, no attempts have been made to apply this to the prediction of human body composition. At the aim of obtaining higher prediction accuracy, stronger generalization ability and faster convergence speed, we developed a modified adaptive genetic algorithm for improving the prediction of human body composition. This algorithm is used to find the optimal weight set for the prediction model, combining the advantages of both the roulette strategy and the optimal retention policies.
(b) It is worth mentioning that the construction of the fitting model also has an important impact on the accuracy of the prediction. Furthermore, its construction depends on the selection of the human characteristic parameters. Therefore, an improved human body composition feature selection algorithm was proposed in this paper. The related background information is discussed in the next section.
(c) In this paper, the suggested feature selection algorithm was firstly used to select the correct feature parameters to build a fitting model. Secondly, we utilized the proposed modified adaptive genetic algorithm to solve the unknown weight set of the model. Finally, a series of simulations were designed to verify the performance of the proposed algorithms.

**Reviewer#2, Concern # 2:** Recent Research: More recent references need to be added and discussed in the literature section and background information about existing approaches in the context of proposed work addressed in this paper:

https://www.sciencedirect.com/science/article/abs/pii/S1568494618306422

https://link.springer.com/article/10.1007%2Fs00500-018-3177-y

https://www.sciencedirect.com/science/article/abs/pii/S1568494615001209

https://link.springer.com/article/10.1007/s11227-018-2279-8

**Author response:** Thank you for pointing out this problem. We read, added and discussed these references in the paragraphs 4 of the introduction section.

**Author action:** We updated the manuscript by reading, citing and discussing these references. The added content is as follows:

(a) As we all know, genetic algorithm is often used for optimization problems in many fields [17], which also have the disadvantages of being easily trapped in local optimization and poor optimization efficiency.
(b) Sangaiah et al. [18] proposed a Hybrid Taguchi-Genetic Learning Algorithm (HTGLA) in which the Taguchi method is inserted between the crossover and mutation operations. The experimental results show that the algorithm can improve prediction accuracy.
(c) Rahmani Hosseinabadi et al. [19] described an extended genetic algorithm which could gain better solutions in terms of computational times and objective values.
(d) The fast genetic algorithm suggested by Khanduzia et al. [20] has higher accuracy and faster running speed, which is a combination of genetic algorithm and fast branch and cut method.

**Reviewer#2, Concern # 3:** Open issues, limitations of the existing approaches or prevalent trend /issues of Predicting human body composition using a modified adaptive genetic algorithm can be elucidated.

**Author response:** Thank you for pointing out this problem. The limitations of the existing approaches have been elucidated in the paragraphs 7 of the introduction section. What is more, we elucidated the prevalent trend of predicting human body composition using a modified adaptive genetic algorithm in the paragraphs 7 of the introduction section.

**Author action:** We updated the manuscript by elucidating the prevalent trend of Predicting human body composition using a modified adaptive genetic algorithm in the paragraphs 7 of the introduction section. The added and modified content is as follows:

Notably, modified adaptive genetic algorithms can not only solve the problem of poor adaptability for these models, but also prevent falling into local optimum. More considerably, although surprisingly it has stronger optimization capability and high convergence efficiency, no attempts have been made to apply this to the prediction of human body composition. At the aim of obtaining higher prediction accuracy, stronger generalization ability and faster convergence speed, we developed a modified adaptive genetic algorithm for improving the prediction of human body composition. This algorithm can be used to find the optimal weight set for the prediction model, combining the advantages of both the roulette strategy and the optimal retention policies.

**Reviewer#2, Concern # 4:** Add the contents in the abstract of the paper and highlight the impact of the proposed work.

**Author response:** Thank you for pointing out this problem. We added the contents in the background, results and conclusion of the abstract to highlight the impact of the proposed work. Please see the abstract in the manuscript.

**Author action:** We updated the manuscript by adding the contents in the abstract of the paper.

**Reviewer#2, Concern # 5:** The method/approach in the context of proposed work should be write in detail.

**Author response:** Thank you for pointing out this problem. This method consists of two parts in this paper. One part is a feature selection algorithm combining improved RReliefF and mRMR, which is used to select the feature parameters that are most suitable for predicting PBF. The improved RReliefF algorithm combines sample distance metric and sample morphometry and its specific improvement steps have been shown in detail in the “Improved RReliefF algorithm” section of the manuscript. In addition, the flowchart and detailed steps of selecting feature parameters of human body composition by using feature selection algorithm have been shown in the “A body composition feature parameter selection method combining improved RReliefF and mRMR” section of the manuscript. The second part is a modified adaptive genetic algorithm for solving unknown weights of a fitted model. We mainly improve the selection operator of the algorithm and the detailed method steps for its improvement are shown in the “Improved selection operator combining roulette selection strategy with optimal retention strategy” section of the manuscript. Using MAGA to solve the unknown weights of a fitted model includes coding, initialization, selection, crossover, and mutation steps and its detailed method steps have been shown in the “The flow of the MAGA-based human body composition prediction algorithm” section of the manuscript.

**Reviewer#2, Concern # 6:** Real time case studies might be add more importance of this chapter. The authors should add the case study and its results.

**Author response:** Thank you for pointing out this problem. We added the case study and its results in the results and discussion section.

**Author action:** We updated the manuscript by adding the case study. The added cases and their results are as follows:

(a) We added a case study of algorithm parameter selection, as shown in Figure 8. Fig 8 demonstrates the different ratios of $\alpha$ to $\beta$ affect PBF prediction errors and the mean square error of the prediction model is the smallest when the ratio of $\alpha$ to $\beta$ is 2: 1.

**Fig 8. Mean square error of different ratios of** $\alpha$ **and****.**

(b) we have added case study comparing MAGA model with support vector regression algorithm (SVR) , artificial neural network algorithm (ANN) and traditional regression method (TRM) models. Please see “Experimental Results and Discussion” section in the manuscript for discussion of its results. The simulation results are as follows:

**Fig 11. Comparison of the actual values and predicted values obtained by different models.**

**Fig 12. Comparison of relative errors between actual values and predicted values obtained by different models.**

**Table 4. Comparison of the training time for different models.**

| Model | Maximum fitness degree | Training time(s) |
| --- | --- | --- |
| GA | 0.5642 | 26.82 |
| AGA | 0.8074 | 28.23 |
| LAGA | 0.9532 | 28.94 |
| MAGA | 0.9921 | 28.58 |
| SVR | - | 30.67 |
| ANN | - | 29.89 |
| TRM | - | 25.46 |

**Table 5. Comparison of the performance of different models.**

| Model | Average relative error (%) | Mean square error | Correlation coefficient | Running time(s) |
| --- | --- | --- | --- | --- |
| AGA | 0.2185 | 6.27 | 0.684 | 2.66 |
| LAGA | 0.1045 | 2.72 | 0.929 | 2.97 |
| MAGA | 0.0500 | 1.30 | 0.982 | 2.84 |
| SVR | 0.0873 | 2.56 | 0.927 | 3.02 |
| ANN | 0.1058 | 2.85 | 0.920 | 2.95 |
| TRM | 0.2348 | 6.73 | 0.596 | 2.49 |

**Reviewer#2, Concern # 7:** Result and discussion should be rewritten to summarize the findings/significance of the work. Please discuss the research limitations and future works in the conclusion.

**Author response:** Thank you for pointing out this problem. We have rewritten the result and discussion to summarize the findings and the significance of the work. Please see the corresponding section in the manuscript. What is more, the research limitations and future works were discussed in the conclusion.

**Author action:** We updated the manuscript by rewriting the result and discussion and discussing the research limitations and future works in the conclusion. The added discussion in the conclusion is as follows:

In future research work, we will consider how to more effectively reduce the time complexity of the algorithm and cross-validation will be used to test model performance in future research.

**Reviewer#2, Concern # 8:** To explore comparative results of existing approaches/methods with relate to the proposed work.

**Author response:** Thank you for pointing out this problem. To explore comparative results of existing approaches with relate to the proposed work, we added simulations comparing the proposed prediction model with the models based on support vector regression algorithm (SVR), artificial neural network algorithm (ANN), traditional regression method (TRM).

**Author action:** We updated the manuscript by adding simulations in the results and disscusion section. The comparative results of existing approaches with relate to the proposed work are as follows:

**Fig 11. Comparison of the actual values and predicted values obtained by different models.**

**Fig 12. Comparison of relative errors between actual values and predicted values obtained by different models.**

**Table 4. Comparison of the training time for different models.**

| Model | Maximum fitness degree | Training time(s) |
| --- | --- | --- |
| GA | 0.5642 | 26.82 |
| AGA | 0.8074 | 28.23 |
| LAGA | 0.9532 | 28.94 |
| MAGA | 0.9921 | 28.58 |
| SVR | - | 30.67 |
| ANN | - | 29.89 |
| TRM | - | 25.46 |

**Table 5. Comparison of the performance of different models.**

| Model | Average relative error (%) | Mean square error | Correlation coefficient | Running time(s) |
| --- | --- | --- | --- | --- |
| AGA | 0.2185 | 6.27 | 0.684 | 2.66 |
| LAGA | 0.1045 | 2.72 | 0.929 | 2.97 |
| MAGA | 0.0500 | 1.30 | 0.982 | 2.84 |
| SVR | 0.0873 | 2.56 | 0.927 | 3.02 |
| ANN | 0.1058 | 2.85 | 0.920 | 2.95 |
| TRM | 0.2348 | 6.73 | 0.596 | 2.49 |

From the comparative results, it can be known that the proposed model has higher accuracy than SVR, ANN and TRM models, and is more efficient than SVR and ANN models.
