# Supplementary material for: Predicting human body composition using a modified adaptive genetic algorithm with a novel selection operator
Source: PLoS One. 2020 Jul 16;15(7):e0235735. doi: 10.1371/journal.pone.0235735 (PMC7365447; doi:10.1371/journal.pone.0235735)
Supplement: Attachment — Submitted filename: Response to Reviewers.docx [file pone.0235735.s003.docx]

**Manuscript Number:** PONE-D-19-20446R1

**Original Article Title: “Predicting human body composition using a modified adaptive genetic algorithm** ”

**To:** PLOS ONE Editor

**Re:** Response to reviewers

Dear Editor,

Thank you for allowing a resubmission of our manuscript, with an opportunity to address the reviewers’ comments.

We are uploading (a) our point-by-point response to the comments (below) (response to reviewers), (b) an updated manuscript with yellow highlighting indicating changes, and (c) a clean updated manuscript without highlights.

We tried our best to improve the manuscript and made some changes in the manuscript. These changes will not influence the content and framework of the paper. We appreciate for Editors/Reviewers’ warm work earnestly, and hope that the correction will meet with approval.

Best regards,

<Bo Chen> et al.

**Reviewer #3, Concern #1:** Why the author has chosen the modified adaptive genetic algorithm predicting human body composition? As there are several algorithms available that can predict better results.

**Author response:** Thank you for pointing out this problem. There are three reasons for choosing modified adaptive genetic algorithm to predict human body composition. First of all, the solution problem of the predictive fitting model of human body composition belongs to the optimal solution problem, and the modified adaptive genetic algorithm can solve this problem well. Secondly, many algorithms are prone to premature convergence when solving the optimal solution, which leads to low prediction precision. The improved selection operator, adaptive crossover operator and mutation operator of the modified adaptive genetic algorithm all have the effect of avoiding premature convergence, which can make the body composition prediction model have higher fitness and accuracy. Finally, according to the training time and running time in table 4 and table 5 in the results and discussion section, it is known that the human body composition prediction model based on the modified adaptive genetic algorithm has higher efficiency. Therefore, on the basis of comprehensive consideration of the overall effect of model fitness, prediction accuracy and efficiency, we chose modified adaptive genetic algorithm to predict human body composition.

**Reviewer #3, Concern #2:** Needs more explanation of Table.2 as well as Table.3 parameters in the result and discussion section.

**Author response:** Thank you for pointing out this problem. Due to the writing error, R_1_~R_8_ in tables 2 and 3 was mistakenly written as R1~R8. In table 2 and table 3, G, A, W, H, PBF and R_1_~R_8_ represent the variables of gender, age, weight, height, percentage of body fat and the human body eight-segment impedance. In addition, in the human body eight-segment impedance, R_1_~R_8_ represents the impedance of the human body's left upper limb, upper trunk, right upper limb, left trunk, right trunk, lower trunk, left lower limb and right lower limb respectively.

**Author action:** We updated the manuscript by correcting the writing error and adding the explanation of tables 2 and 3 parameters in the results and discussion section.

**Reviewer #3, Concern #3:** In Table.4 the authors present the comparison of the training time for different models but how actually derived the Maximum fitness degree and Training time(s) values are unclear. Elaborate with suitable examples. Data seems to fabricate.

**Author response:**

Thank you for pointing out this problem. Our data were obtained through experiments, and the software can record the corresponding maximum fitness and training time. Taking the human body composition prediction model based on modified adaptive genetic algorithm proposed in this paper as an example, the experimental process and reasoning process are detailed as follows.

Step 1 Inputing training data, preprocessing data, and coding and initialization of modified adaptive genetic algorithm.

Step 2 Calculate the fitness function of individuals and the formula is: $fit(k)=\frac{1}{1+object(k)}$, where $object(k)=\frac{1}{m}\sum_{i=1}^{m} \frac{\left|f_{k}(\omega)_{i}-F_{i}\right|}{F_{i}}$，$object(k)\in[0\text{，+}\infty)$，and $fit(k)\in(0,1]$.

Step 3 The improved selection operator, adaptive crossover operator, and mutation operator of the modified adaptive genetic algorithm are executed respectively to iteratively evolve the population.

Step 4 Determine termination conditions. When the error between the predicted and actual values is less than or equal to 0.01, or when the number of iterations reaches a maximum value of 400, iteration is terminated. If one of the termination conditions is met, the program is stopped and the maximum fitness, the optimal unknown weight subset, and the program running time are output, otherwise the fitness is recorded, Gen = Gen + 1 and returns to Step 2.

In the above process, since the final condition of the number of iterations Gen = 400 was reached in our experiments, the maximum fitness of the output is the largest of the 400 fitness. In addition, the “clock” and “etime” functions in MATLAB are used to record the running time of the program, and the running time of the program is the training time. It is worth emphasizing that the data of other models are also obtained through software records.

**Reviewer #3, Concern #4:** Very hard to find the novelty of this manuscript? The authors are suggested highlighting their own contributions.

**Author response:** Thank you for pointing out this problem. The novelty of this manuscript has two aspects and the novelty and contribution of this manuscript have been expounded in the last paragraph of the introduction part. The novelty and contribution are elaborated further here.

(1) In order to obtain a subset of feature parameters with large correlation and small redundancy, an improved feature selection algorithm combining improved RReliefF algorithm and mRMR algorithm was proposed. Firstly, the RReliefF algorithm was improved to construct the sample similarity distance model, taking into account the Euclidean distance and morphological distance of the samples. Secondly, combining with the improved RReliefF and mRMR algorithm, the feature subset used to construct the predictive fitting model of human body composition was screened out.
(2) In order to avoid premature convergence of the algorithm, a modified adaptive genetic algorithm was proposed. Firstly, the selection operator of the adaptive genetic algorithm was improved by combining the optimal selection strategy and roulette strategy. Secondly, the modified algorithm was used to solve the human body composition prediction model, which improves the accuracy of human body composition prediction.

**Reviewer #3, Concern #5:** If possible explain briefly the dataset and parameters which have been used in this article.

**Author response:** Thank you for pointing out this problem. The data set used in this paper consists of sample data from 220 healthy Chinese, including 96 females and 124 males. The parameters included in the data set are G, A, W, H, PBF and R_1_~R_8_, which respectively represent the variables of gender, age, weight, height, percentage of body fat and human body eight-segment impedance. What’s more, R_1_~R_8_ represents the impedance of the human body's left upper limb, upper trunk, right upper limb, left trunk, right trunk, lower trunk, left lower limb and right lower limb respectively.

**Author action:** We updated the manuscript by adding the explanation of the data and parameters in the results and discussion section.
