# Supplementary material for: Predicting human body composition using a modified adaptive genetic algorithm with a novel selection operator
Source: PLoS One. 2020 Jul 16;15(7):e0235735. doi: 10.1371/journal.pone.0235735 (PMC7365447; doi:10.1371/journal.pone.0235735)
Supplement: Attachment — Submitted filename: Response to Reviewers.docx [file pone.0235735.s004.docx]

**Manuscript Number:** PONE-D-19-20446R2

**Article Title: “Predicting human body composition using a modified adaptive genetic algorithm with a novel selection operator**”

**To:** PLOS ONE Editor

**Re:** Response to reviewers

Dear Editor,

Thank you for allowing a resubmission of our manuscript with the opportunity to address the reviewers’ comments.

We are uploading: (a) our point-by-point response to the comments from the reviewers and editor (below); (b) an updated manuscript, where yellow highlighting indicating changes; and (c) a clean, updated manuscript without highlights.

We have tried our best to improve the manuscript. The changes we have made will not influence the content or framework of the paper. We sincerely appreciate the work of the editor and reviewers and hope that our corrections will meet with approval.

Best regards,

<Bo Chen> et al.

**Editor, Concern #1:** The authors should ask a native English speaker or consult a professional English editing service to proofread the paper. Please upload the certificate of language correction in the submission. Some examples, to name a few:

-- Abstract: "Background: Changes to human body composition reflect, to some extent, changes in health status."  (Incomplete sentence).

-- Introduction: "However, once the regression equation is determined, it becomes difficult to adjust it according to the situation, meaning adaptability is poor." (Incomplete sentence).

-- Improved RReliefF algorithm: "Where ik x is the k-th body physiological parameter value of sample"  (where instead of Where).

**Author response:** Thank you for pointing out these errors. We have tried our best to check and modify such errors in the manuscript, including misspellings, grammatical errors, inappropriate expressions and so on. Additionally, we engaged the services of a professional English editing service to proofread the paper and will upload the certificate of language correction in the submission. There are many changes which have been highlighted in yellow in the manuscript.

**Author action:** We set out correct the errors in the article and so improve the English.

**Editor, Concern #2:** Equations need to be re-typed. The current version lacks of clear formulae and equations. All equations must be numbered.

**Author response:** Thank you for pointing out this problem. We have re-entered all formulas to meet the required version and all equations have now been numbered. Changes are highlighted in yellow.

**Author action:** We have updated the manuscript by re-entering and numbering all the formulas.

**Editor, Concern #3:** Please append statistical analysis for the experimental results in Tables 4-5 to prove the achieved results being statistically significance.

**Author response:** Thank you for pointing out this issue. Table 4 shows only the maximum fitness and training time of each model. The maximum fitness is calculated by the fitness function. Since the TRM, SVR and ANN algorithms do not involve fitness calculations, they do not have a figure for maximum fitness. The training time term refers to the prediction model training time. Therefore, statistical analysis is not covered in Table 4. In addition, since the other items in Table 5 do not involve statistical analysis, we only made a statistical analysis of the correlation coefficients in that table. The correlation coefficient refers to the correlation between the predicted value and the model’s true value. We used a two-tailed test of statistical analysis for the correlation coefficient. Statistical analysis showed that the correlation coefficients between the predicted and true values of the six models were all greater than 0.5 and the P values (significance level) are all far less than 0.01, showing that they are strongly correlated and the results are statistically significant. Additionally, we added statistical analysis results to Table 5 (as shown below), and the corresponding analysis is supplied in the section ‘Experimental Results and Discussion’.

**Table 5. Comparison of the performance of different models.**

| Model | Mean relative error (%) | Mean square error | Correlation coefficient | Running time(s) |
| --- | --- | --- | --- | --- |
| AGA | 0.2185 | 6.27 | 0.684^a^ | 2.66 |
| LAGA | 0.1045 | 2.72 | 0.929^b^ | 2.97 |
| MAGA | 0.0500 | 1.30 | 0.982^c^ | 2.84 |
| SVR | 0.0873 | 2.56 | 0.927^d^ | 3.02 |
| ANN | 0.1058 | 2.85 | 0.920^e^ | 2.95 |
| TRM | 0.2348 | 6.73 | 0.596^f^ | 2.49 |

a.	P value (significance level) is 0.001, which is far less than 0.01.

b.	P value (significance level) is approximately 0, which is far less than 0.01.

c.	P value (significance level) is approximately 0, which is far less than 0.01.

d.	P value (significance level) is approximately 0, which is far less than 0.01.

e.	P value (significance level) is approximately 0, which is far less than 0.01.

f.	P value (significance level) is 0.006, which is far less than 0.01.

**Author action:** We updated the manuscript by including a statistical analysis of the correlation coefficients in Table 5 and supplementing the statistical analysis.

**Editor, Concern #4:** Future works must be appended by adding 3-5 more solid works from this sentence: "In future research work, we will consider how to more effectively reduce the time complexity of the algorithm and cross-validation will be used to test model performance in future research”.

**Author response:** Thank you for pointing out this issue. The future works have been appended by adding three solid works: 1) the influence of different sample sizes on the prediction effect will be considered; 2) we will explore the predictive effects of the proposed algorithm on more body components such as total body water and body fat; 3) the combination of the two intelligent algorithms will be considered to solve the unknown weights in the body composition fitting model.

**Author action:** We updated the manuscript by adding three solid works in future works. The updated sentence reads as follows:

We will conduct further research in the future based on the following five aspects: 1) we will consider how to more effectively reduce the time complexity of the algorithm; 2) cross-validation will be used to test model performance; 3) the influence of different sample sizes on the prediction effect will be considered; 4) we will explore the predictive effects of the proposed algorithm on more body components such as total body water and body fat; 5) the combination of the two intelligent algorithms will be considered to solve the unknown weights in the body composition fitting model.

**Editor, Concern #5:** The coherence to PLOS ONE should be explained clearly in the Introduction and through recent related articles.

**Author response:** Thank you for pointing this problem out. We have rearranged recent related articles and revised the introduction to make our article clearly coherent. Modified sections are highlighted in yellow.

**Author action:** We have updated the manuscript by combing and trimming the introduction.

**Editor, Concern #6:** The authors should consider to change the paper title to reflect more contribution and coherence to PLOS.  "Predicting human body composition using a modified adaptive genetic algorithm." What is modified here?

**Author response:** Thank you for pointing out this problem. We have improved the selection operator of the adaptive genetic algorithm by combining both roulette selection and optimal retention strategies. This combined strategy retains the most adaptive individuals while removing the least suitable individuals, ensuring a diversity of choice. To reflect more contribution and coherence to PLOS, our revised title is “Predicting human body composition using a modified adaptive genetic algorithm with a novel selection operator”.

**Author action:** We updated the manuscript by changing our title to “Predicting human body composition using a modified adaptive genetic algorithm with a novel selection operator”.

**Reviewer #3, Concern #1:** Author should highlight the contribution.

**Author response:** Thank you for pointing out this error. We highlighted the contribution of this article in paragraphs 9 to 14 of the ‘Introduction’ section. Contributions are as follows:

There are three novel contributions in this paper. The suggested feature selection algorithm was firstly used to select the correct feature parameters to build a fitting model. Secondly, we utilized the proposed modified adaptive genetic algorithm to solve the unknown weight set of the model. Finally, a series of simulations were designed to verify the performance of the proposed algorithms. The main details of these contributions are:

(1) We propose an improved RReliefF algorithm which combines sample distance metric with sample morphometry and develop a sample similarity distance model, which combines human numerical parameter sample distance and sample morphological distance.

(2) Based on the improved human body composition feature selection algorithm which combines RReliefF and mRMR, the detailed flow of the algorithm’s selected feature is designed to obtain the preferred feature set of human body composition.

(3) We propose an improved selection operator which combines both roulette selection and optimal retention strategies. This combined strategy retains the most adaptive individuals while removing the least suitable individuals, ensuring a diversity of individual choices.

(4) A flow chart of this human body composition prediction algorithm based on a modified adaptive genetic algorithm is developed, and the weight-solving problem of the human body component prediction model is solved.

(5) We designed algorithm simulations to analyse how to choose the algorithm parameters, algorithm adaptability and efficiency as well as the proposed algorithm’s prediction accuracy of the body composition model.

**Author action:** We updated the manuscript by highlighting the contribution of this article in the ‘introduction’ section.

**Reviewer #3, Concern #2:** More emphasize on the dataset. Sources of dataset should required to mention.

**Author response:** Thank you for pointing out this issue. We added more explanation to the data set and its source. The dataset contains the physiological characteristic parameters of 220 healthy Chinese participants, including 96 females and 124 males, who ranged in age from nine to 84, in height from 149 to 190cm, and in weight from 38.4 to 121.4kg. The dataset came from a hospital (the Haidian Maternal and Child Health Hospital) in Beijing, China. We have added supplementary information about the dataset to the section ‘Experimental data’.

**Author action:** We updated the manuscript by adding supplementary information about the dataset to the section ‘Experimental data’.

**Reviewer #3, Concern #3:** More explanations on the Figures and Tables.

**Author response:** Thank you for pointing this out. We have added more explanations to Tables 4-5 and Figures 4, 7 and 8. These changes are highlighted in yellow in the section ‘Experimental Results and Discussion’.

(1) The addition of Table 4 is explained as follows:

Since TRM, SVR and ANN algorithms don’t involve the calculation of fitness function, there are no maximum fitness degrees in Table 4.

(2) The additions of Table 5 as well as Figures 4, 7 and 8 are explained as follows

Additionally, the correlation coefficients between the predicted and true values of the six models are all greater than 0.5 and the P values are all far less than 0.01, which shows that they are strongly correlated and the results are of statistical significance. In particular, the correlation coefficient of the proposed model is 0.98 and the P value is 0. In sum, this shows that the predicted value obtained by the proposed model is the closest to the real value and the overall error rate is the lowest of all models, meaning it has the highest prediction accuracy.

(3) The addition of Figure 4 is explained as follows:

To explore the influence of different values of key parameters ( and ) on the prediction accuracy in the feature selection algorithm, the comparison experiment was set.

In addition, according to formula 1.5, $\alpha+\beta=1$. Therefore, the value of $\alpha$ is set to one third and the value of $\beta$ is set to two thirds in the feature selection algorithm to obtain a more satisfying prediction accuracy.

**Author action:** We updated the manuscript by adding more explanations to Tables 4-5 and Figures 4, 7 and 8 in the section ‘Experimental Results and Discussion’.
